# Control of Intestinal Inflammation, Colitis-Associated Tumorigenesis, and Macrophage Polarization by Fibrinogen-Like Protein 2

**DOI:** 10.3389/fimmu.2018.00087

**Published:** 2018-01-30

**Authors:** Ying Zhu, Jie Zhou, Yi Feng, Liying Chen, Longhui Zhang, Fei Yang, Haoran Zha, Xinxin Wang, Xiao Han, Chi Shu, Yisong Y. Wan, Qi-Jing Li, Bo Guo, Bo Zhu

**Affiliations:** ^1^Institute of Cancer, Xinqiao Hospital, Third Military Medical University, Chongqing, China; ^2^Department of Pathogenic Biology, Third Military Medical University, Chongqing, China; ^3^Department of Microbiology and Immunology, School of Medicine, University of North Carolina at Chapel Hill, Chapel Hill, NC, United States; ^4^Department of Immunology, Duke University Medical Center, Durham, NC, United States

**Keywords:** fibrinogen-like protein 2, colitis, colitis-associated colorectal cancer, immune regulation, intestinal inflammation, macrophage polarization

## Abstract

Fibrinogen-like protein 2 (Fgl2) is critical for immune regulation in the inflammatory state. Elevated Fgl2 levels are observed in patients with inflammatory bowel disease (IBD), but little is known about its functional significance. In this study, we sought to investigate the role of Fgl2 in the development of intestinal inflammation and colitis-associated colorectal cancer (CAC). Here, we report that Fgl2 deficiency increased susceptibility to dextran sodium sulfate-induced colitis and CAC in a mouse model. During colitis development, the expression of the membrane-bound and secreted forms of Fgl2 (mFgl2 and sFgl2, respectively) in the colon were increased and predominantly expressed by colonic macrophages. In addition, using bone marrow chimeric mice, we determined that Fgl2 function in colitis is strictly related to its expression in the hematopoietic cells. Loss of Fgl2 induced the polarization of M1, but suppressed that of M2 both *in vivo* and *in vitro*, independent of intestinal inflammation. Thus, Fgl2 suppresses intestinal inflammation and CAC development through its role in macrophage polarization and may serve as a therapeutic target in inflammatory diseases, including IBD.

## Introduction

The maintenance of intestinal homeostasis requires a balance of immune tolerance and effector functions. Disruption of this homeostasis results in pathologies such as inflammatory bowel disease (IBD), of which there are two major clinical forms: ulcerative colitis and Crohn’s disease. There are no effective cures for IBD (1, 2). Importantly, long-term intestinal inflammation is a key risk factor for colitis-associated colorectal cancer (CAC) (3). IBD is characterized by abnormalities in intestinal immunity, specifically, the pro-inflammatory response in the gut (4, 5). Thus, further understanding of the immunopathogenesis of intestinal inflammation can lead to the development of novel therapies for IBD.

During IBD, immune cells, including dendritic cells (DCs), neutrophils, and effector T cells, activate and trigger inflammation by secreting pro-inflammatory cytokines. On the other hand, regulatory T cells (Tregs) suppress the mucosal immune response, although their inhibitory capacity is insufficient to control IBD progression. In particular, colonic macrophages can both promote or inhibit IBD pathogenesis depending on their M1- or M2-polarized phenotype. The classically activated M1 type promotes colitis primarily by secreting pro-inflammatory cytokines, such as interleukin (IL)-6, -1β, and interferon (IFN)-γ, leading to tissue damage. In contrast, the alternatively activated M2 type contributes to the resolution of colitis *via* upregulation of arginase (Arg)-1, mannose receptor (MR), Ym1, IL-10, and Fizz1, which promote tissue repair (6–8). Transferring Tregs or M2 macrophages or inducing M2 polarization has been shown to suppress experimental colitis (9, 10). Thus, activating an anti-inflammatory response in the intestine is an attractive strategy for restoring intestinal homeostasis.

Fibrinogen-like protein 2 (Fgl2) is a member of the fibrinogen superfamily that has well-known roles in the pathogenesis of inflammatory disorders and has multiple functions in immune regulation (11–13). Fgl2 has been shown to inhibit the maturation of bone marrow (BM)-derived DCs and the proliferation of effector T cells, promote macrophage activation, and maintain the immunosuppressive activity of Tregs. It also induces the apoptosis of B cells and macrophages (14–17). We speculated that the immunomodulatory activity of Fgl2 plays an important role in the pathogenesis of IBD. Interestingly, a clinical study reported that Fgl2 expression was increased in patients with active IBD (18). However, a comprehensive analysis of Fgl2 function in IBD pathogenesis is still lacking.

To address this issue, we investigated the contribution of Fgl2 to IBD development by evaluating its expression in the inflamed intestine and examining the effects of Fgl2 deficiency on dextran sodium sulfate (DSS)-induced colitis in mice. We found that Fgl2 is mainly expressed in colonic macrophages in colitis and suppresses DSS-induced colitis and subsequent CAC development. In addition, we demonstrate that Fgl2 regulates macrophage polarization and function, which is associated with the protective effect of Fgl2 in intestinal inflammation.

## Materials and Methods

### Mice

Fibrinogen-like protein 2 knockout mice (hereafter referred to as Fgl2^−/−^) were kindly provided by Dr. Steve Smiley (The Trudeau Institute, NY, USA) and maintained on C57BL/6 genetic background. Their age-matched and sex-matched homozygous wild-type littermates were used as controls. C57BL/6 mice were obtained from the Animal Institute of the Academy of Medical Science (Beijing, China). All mice were kept in laminar flow cabinets under specific pathogen-free conditions at the Animal Center of the Third Military Medical University. The animal study protocols were approved by the Third Military Medical University Institutional Animal Care and Use Committee.

### Induction and Evaluation of Acute Colitis and CAC

Mice (male or female, 8–10 weeks old) were administered drinking water containing 2.5% DSS (MW 36–50 kDa; MP-0216011050; MP Biomedicals, Solon, OH, USA) for 5 or 6 days, followed by regular water up to 9 days. Body weight, rectal bleeding, and stool consistency were monitored daily. A clinical score was assigned based on changes in weight loss, stool consistency, and rectal bleeding (19). Colon tissue samples were fixed in 4% (v/v) paraformaldehyde, embedded in paraffin, and cut into 4-µm sections that were stained with hematoxylin and eosin. Histological scoring was performed to quantify epithelial damage and inflammatory cell infiltration (20). The severity of colitis was assessed by a pathologist blinded to the treatment groups. To induce CAC, 8- to 10-week-old female mice were intraperitoneally injected with 10 mg/kg azoxymethane (AOM) on day 0. After 7 days, the mice were treated with 1.8% DSS for 5 days, followed by regular drinking water for 16 days. Body weight was monitored daily. The mice were sacrificed after three cycles of DSS administration, and tumor number and size (length × width) were determined.

### Western Blot Analysis

Mouse colonic tissue was harvested at predetermined time points and proteins were extracted with radioimmunoprecipitation assay buffer composed of 1% Triton X-100, 0.5% Na-deoxycholate, 0.1% sodium dodecyl sulfate, 20 mmol/l Tris–HCl (pH 7.4), and 1% phenylmethylsulfonyl fluoride (Beyotime Institute of Biotechnology, Shanghai, China). Equal amounts of protein sample were resolved on a 12% sodium dodecyl sulfate-polyacrylamide gel and transferred to a polyvinylidene difluoride membrane. After blocking with 5% non-fat milk, the membrane was probed with primary and secondary antibodies, and immunoreactivity was detected by enhanced chemiluminescence (Pierce, Rockford, IL, USA). Anti-mouse Fgl2 antibody (clone 6D9, 1:1,000 dilution; Abnova, Taiwan) and anti-mouse β-actin (clone AC-15, 1:2,000 dilution; Sigma-Aldrich, St. Louis, MO, USA) were the primary antibodies. Goat anti-mouse IgG (1:5,000 dilution; Beyotime Institute of Biotechnology) was used as a secondary antibody. The gray values of the western blotting bands were procured using ImageJ software.

### Immunofluorescence Analysis and Confocal Microscopy

Frozen sections of mouse colonic tissue were fixed with ice-cold acetone for 10 min at −20°C. After washing with phosphate-buffered saline (PBS), the sections were incubated with blocking solution (5% bovine serum albumin in PBS) for 30 min at 37°C and then incubated overnight at 4°C with the following antibodies: mouse anti-mouse Fgl2 (clone 6D9, 1:100 dilution; Abnova); and rat anti-mouse F4/80 (clone CI:A3-1, 1:100 dilution), Armenian hamster anti-mouse cluster of differentiation (CD)11c (clone N481, 1:100 dilution), and rabbit polyclonal anti-mouse CD31 (1:100) (all from Abcam, Cambridge, MA, USA). After washing four times with PBS, the sections were incubated for 30 min at room temperature with the following secondary antibodies: Cy3-labeled goat anti-mouse IgG (1:200 dilution), Alexa Fluor 488-labeled goat anti-rabbit IgG (1:200 dilution), or Alexa Fluor 488-labeled goat anti-rat IgG (1:200 dilution) (all from Beyotime Institute of Biotechnology); or Alexa Fluor 488-labeled goat anti-Armenian hamster antibody (1:100 dilution; Santa Cruz Biotechnology, Santa Cruz, CA, USA). After counterstaining with 4′,6-diamidino-2-phenylindole dihydrochloride (Beyotime Institute of Biotechnology), the sections were observed under a confocal microscope (TCS SP5 X; Leica, Wetzlar, Germany).

### Generation of BM Chimeric Mice

Bone marrow cells were flushed from the tibia and femur of WT and Fgl2^−/−^ mice (6–8 weeks old), and erythrocytes were removed using RBC Lysis Buffer (Biolegend, San Diego, CA, USA). Recipient mice (6–8 weeks old) were irradiated at 1,100 rad, and BM cells resuspended in 0.1 ml of 0.9% sodium chloride were intravenously injected *via* the retro-orbital plexus (5 × 10^6^ cells/mouse). Chimeric mice were administered antibiotic water (800 mg/ml gentamycin) for 1 week after BM transplantation and housed for 8 weeks before induction of colitis by DSS treatment. To confirm BM reconstitution, CD45^+^ allelic variants of blood leukocytes from chimeric mice were detected by flow cytometry using antibody against CD45.1 (clone A20, 1:200) or CD45.2 (clone 140, 1:200) (both from Biolegend). Cells were sorted on a Canto II cytometer (BD Biosciences, Franklin Lakes, NJ, USA), and data were analyzed with FlowJo-X software (Tree Star, Ashland, OR, USA).

### Isolation of Colonic Lamina Propria (cLP) Cells by Flow Cytometry

Mice were sacrificed on day 6 or 9 after DSS administration. The colon was cut longitudinally, washed in ice-cold PBS, and cut into pieces that were incubated in pre-digestion solution composed of Hank’s Balanced Salt Solution containing 5% fetal bovine serum (FBS; Gibco, Grand Island, NY, USA), 5 mM EDTA, and 1 mM dithiothreitol for 30 min at 37°C. Enzymatic digestion was performed in Iscove’s Modified Dulbecco’s Medium (Hyclone, Logan, UT, USA) supplemented with 5% FBS, 1 mg/ml collagenase D (Roche Diagnostics, Indianapolis, IN, USA), and 0.5 mg/ml DNase I (Roche Diagnostics) for 1 h at 37°C with gentle shaking. The cell suspension was passed through a 100-µm nylon mesh and centrifuged. cLP cells were enriched on a 40/80 Percoll gradient (Sigma-Aldrich) (21). To analyze the components and phenotype of inflammatory cells in the colon, cLP cells were incubated with anti-CD16/-CD32 blocking antibody (clone 93) and then labeled with anti-CD11b (clone M1/70), anti-F4/80 (clone BM8), anti-CD206 (clone C068C2), anti-Dectin-1 (clone RH1), anti-CD11c (clone N418), anti-major histocompatibility complex (MHC) II (I-A/I-E, clone M5/114.15.2), and anti-Gr-1 (clone RB6-8C5) antibodies (all from Biolegend). Antibodies were used at 1:100 dilution. The cells were sorted on a Canto II cytometer, and data were analyzed with FlowJo-X software.

### Peritoneal Macrophage (PEM) Isolation and Stimulation

WT and Fgl2^−/−^ mice (8–10 weeks old) were sacrificed and PEMs were isolated by flushing the peritoneal cavity with PBS. Cells were seeded in 12-well dishes in Roswell Park Memorial Institute medium supplemented with 10% FBS and 1% penicillin–streptomycin (Hyclone). Non-adherent cells were removed by extensive washing with PBS and adherent macrophages were treated with 100 ng/ml lipopolysaccharide (LPS; Sigma-Aldrich) or 20 ng/ml mouse recombinant IL-4 (PeproTech, Rocky Hill, NJ, USA) for further analysis.

### Cytokine Enzyme-Linked Immunosorbent Assay (ELISA)

Proteins were extracted from the colon of colitic mice. Samples were weighed and homogenized with PBS containing 1% phenylmethylsulfonyl fluoride. Tumor necrosis factor (TNF)-α, IL-1β, -6, -17A, -10, -4, -17F, -21, -22, transforming growth factor (TGF)-β, and IFN-γ levels were quantified by ELISA using a kit (MLBIO, Shanghai, China) according to the manufacturer’s instructions. For *in vitro* stimulation of macrophage assay, PEMs were isolated, and the adherent macrophages were stimulated with LPS (100 ng/ml) or mouse recombinant IL-4 (20 ng/ml). The culture supernatant was collected 48 h later, and IL-1β and -10 levels in the culture medium were detected by ELISA (MLBIO).

### RNA Isolation and Quantitative Reverse-Transcription PCR

Peritoneal macrophages were isolated and adherent macrophages were stimulated with LPS (100 ng/ml) or mouse recombinant IL-4 (20 ng/ml) for 12 h, and total RNA was extracted from inflamed colonic macrophages of WT and Fgl2^−/−^ mice using TRIzol reagent (Takara Bio, Otsu, Japan) or the Micro Total RNA Isolation kit (Invitrogen, Carlsbad, CA, USA). The RNA was reverse-transcribed with Reverse Transcription Reagent (Takara Bio) according to the manufacturer’s instructions. Gene expression was evaluated by qPCR using a SYBR Green Premix Ex Taq kit (Takara Bio). The primer sequences are shown in Table S1 in Supplementary Material.

### Statistics

Quantitative data are expressed as the mean ± SEM. Data were analyzed using GraphPad Prism version 7 (GraphPad Software, San Diego, CA, USA). Differences between groups were evaluated with either an unpaired two-tailed Student’s *t*-test or analysis of variance with Bonferroni correction. A *P*-value <0.05 was considered statistically significant. All experiments were independently repeated at least three times.

## Results

### Identification of Fgl2 Expression in DSS-Induced Colitis

**Figure 1:**
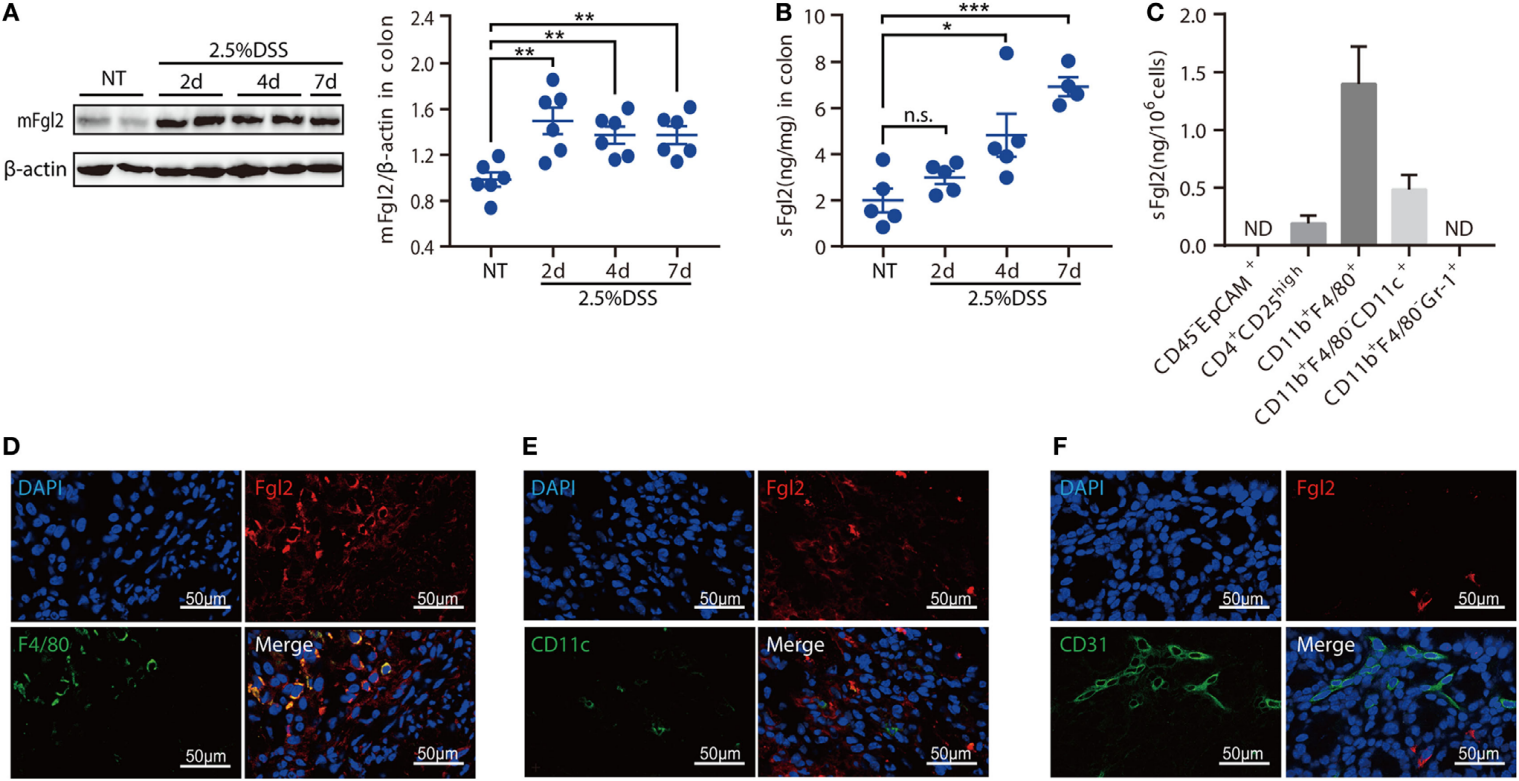
Identification of fibrinogen-like protein 2 (Fgl2)-expressing cells in dextran sodium sulfate (DSS)-induced colitis. **(A,B)** Colitis was induced in C57BL/6 mice. At the indicated time points, mice were sacrificed, and colon tissue samples were obtained and analyzed for Fgl2 expression by western blotting **(A)** and enzyme-linked immunosorbent assay (ELISA) **(B)**. **(C)** CD45^−^ EpCAM^+^, CD4^+^ CD25^high^, CD11b^+^ F4/80^+^, CD11b^+^ CD11c^+^, and CD11b^+^ Gr-1^+^ cells were sorted from colon tissue samples by flow cytometry on day 7, and sFgl2 expression was determined by ELISA. **(D–F)** Representative immunofluorescence micrographs of colonic mucosa from colitic mice showing F4/80^+^ [**(D)**, green], CD11c^+^ [**(E)**, green], CD31^+^ [**(F)**, green], and mFgl2^+^ [panels **(D–F)**, red] cells. Colocalization of mFgl2 with the macrophage or dendritic cell marker is shown in the merged images (*n* = 4–6 mice). Data are expressed as the mean ± SEM and are representative of three independent experiments. *P < 0.05, **P < 0.01, ***P < 0.001, n.s., not significant (as determined by one-way analysis of variance followed by Bonferroni correction).

Given the potential importance of Fgl2 in the pathogenesis of IBD, we first investigated the expression pattern of Fgl2 in the colon under steady state and under inflammatory condition induced by DSS, a widely used chemical irritant that induces intestinal inflammation with clinical and histological features of human IBD (22). Fgl2 exists in a membrane-bound and a secreted form (mFgl2 and sFgl2, respectively) (11). We found that the expression of both forms was increased by DSS treatment (Figures 1A,B). We also noted a gradual upregulation of sFgl2 expression along with colitis progression (Figure 1B; Figures S1A,B in Supplementary Material). sFgl2 is mainly secreted by T cells, specifically, activated Tregs (23); however, whether other types of cells secrete sFgl2 under colitis condition remains unclear. To address this, we sorted colonic epithelial cells (CD45^−^ EpCAM^+^), Tregs (CD4^+^ CD25^high^), macrophages (CD11b^+^ F4/80^+^), DCs (CD11b^+^ F4/80^−^ CD11c^+^), and neutrophils (CD11b^high^ Gr-1^high^) on day 7 after DSS induction (Figure S1C in Supplementary Material) and analyzed sFgl2 production by ELISA. sFgl2 was detected in Tregs and DCs but not in epithelial cells or neutrophils. Notably, we found that macrophages—a dominant immune cell type in cLP that are important in IBD pathogenesis—expressed high levels of sFgl2 (Figure 1C). mFgl2 is expressed by several cell types, including macrophages, DCs, and endothelial cells (10, 16, 17). To readily visualize the cells expressing Fgl2 upon DSS exposure, we carried out a colocalization analysis by confocal microscopy and found that macrophages (F4/80^+^ cells) (Figure 1D) and a fraction of DCs (CD11c^+^ cells) (Figure 1E), but not endothelial cells (CD31^+^ cells), expressed mFgl2 (Figure 1F). Together, these data suggest that Fgl2 is induced in the inflamed colon and that it is mostly expressed by various immune cells types, specially, macrophages, in DSS-induced colitis.

### Fgl2 Deficiency Aggravates DSS-Induced Colitis

**Figure 2:**
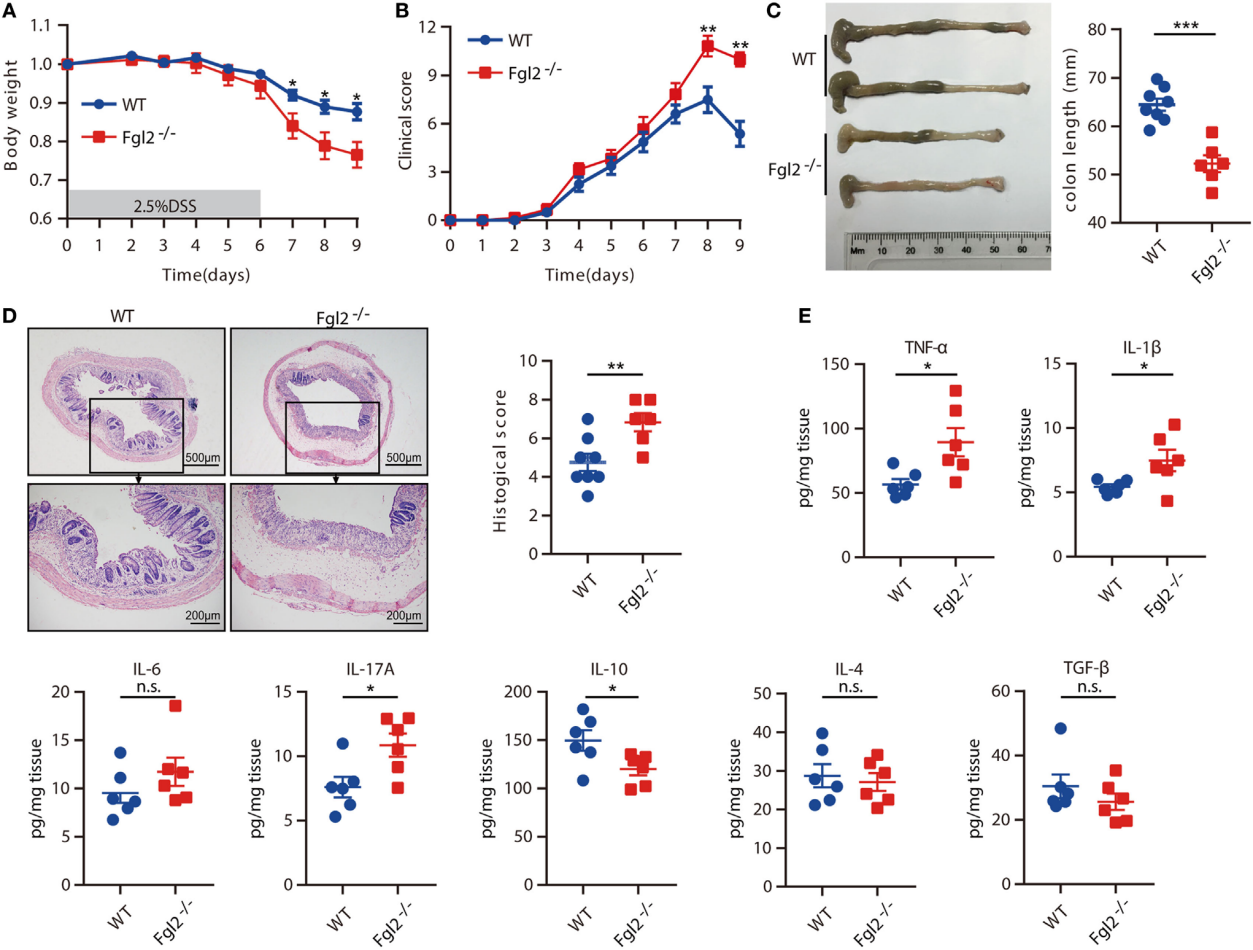
Loss of fibrinogen-like protein 2 (Fgl2) aggravates dextran sodium sulfate (DSS)-induced colitis. Fgl2^−/−^ mice and WT littermates were administered 2.5% DSS in drinking water for 6 days, followed by normal drinking water for 3 days. **(A)** Change in weight over time is expressed as the percentage of the initial body weight (*n* = 8 for WT, *n* = 6 for Fgl2^−/−^). **(B)** Clinical score (*n* = 8 for WT, *n* = 6 for Fgl2^−/−^). **(C)** Colon length (*n* = 8 for WT, *n* = 6 for Fgl2^−/−^). **(D)** Representative hematoxylin and eosin-stained images of colon tissue samples from indicated groups of mice and corresponding histological score on day 9 of DSS induction (*n* = 8 for WT, *n* = 6 for Fgl2^−/−^). **(E)** On day 9, pro-inflammatory and regulatory cytokine production in the colonic mucosa was evaluated by enzyme-linked immunosorbent assay (*n* = 6 per group). Data are representative of three independent experiments. Quantitative data are shown as the mean ± SEM. The statistical significance of differences was determined by two-way analysis of variance with Bonferroni correction **(A,B)** and unpaired two-tailed Student’s *t*-test **(C–E)**. *P < 0.05, **P < 0.01, ***P < 0.001, n.s., not significant.

To assess the significance of Fgl2 expression in colitis, we challenged Fgl2^−/−^ and their WT littermates (controls) with DSS and then monitored their susceptibility to colitis. Upon DSS administration, Fgl2^−/−^ mice exhibited greater weight loss than their WT counterparts (Figure 2A). Fgl2^−/−^ mice also showed more severe colitis symptoms, including abundant diarrhea and rectal bleeding, resulting in higher clinical scores (Figure 2B). Colon length was measured to determine the extent of colonic injury and we found distinctly shorter colons in the Fgl2^−/−^ mice than in the control mice (Figure 2C). Consistent with these observations, pathological changes in the colon were more severe in Fgl2^−/−^ mice, as evidenced by larger areas of epithelial destruction, increased submucosal edema, and greater inflammatory cell infiltration relative to WT mice, in which tissue architecture was partly preserved and inflammation was limited. Consequently, Fgl2^−/−^ mice had higher histological scores than their WT littermates (Figure 2D). Thus, loss of Fgl2 exacerbates the clinical and histological features of DSS-induced colitis.

Aberrant cytokine production is closely linked to intestinal inflammation and the clinical symptoms of IBD (4). Therefore, we investigated whether loss of Fgl2 affects cytokine profiles after DSS treatment by measuring cytokine levels in colonic tissue on day 9 of DSS induction. Fgl2 deficiency was associated with a marked increase in the levels of the pro-inflammatory cytokines, including TNF-α, IL-1β, IL-17A, and IFN-γ, whereas the levels of IL-6, IL-17F, IL-21, and IL-22 remained unaffected. Conversely, the production of the regulatory cytokine IL-10 was decreased in Fgl2^−/−^ as compared with WT mice upon DSS induction, while IL-4 and TGF-β production were largely unaltered (Figure 2E; Figure S2 in Supplementary Material). Thus, Fgl2 deficiency also promotes the inflammatory response in DSS-induced colitis.

Taken together, these results demonstrate that Fgl2 is critical for suppressing DSS-induced colitis.

### Hematopoietic Cell-Derived Fgl2 Is Involved in Limiting Intestinal Inflammation

**Figure 3:**
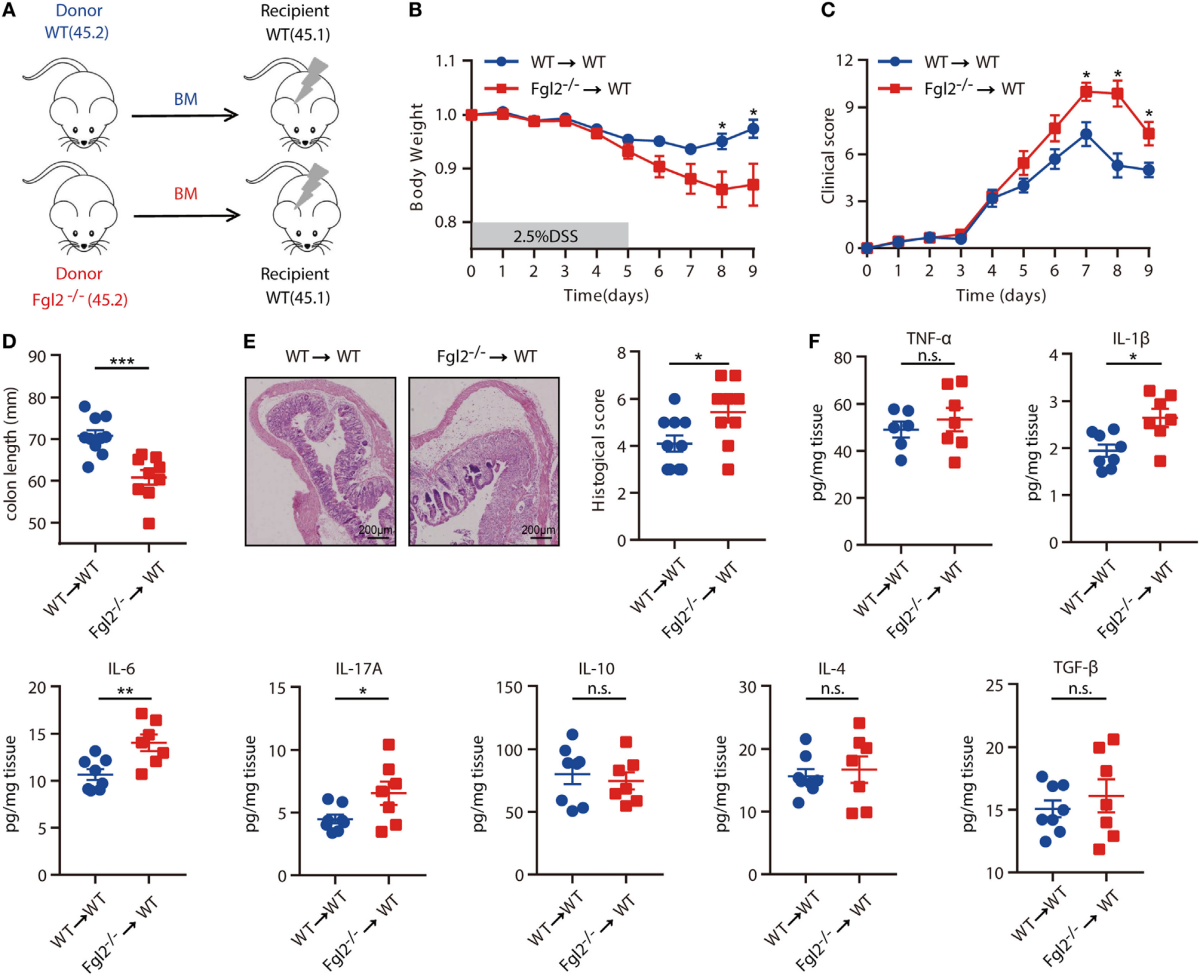
Fibrinogen-like protein 2 (Fgl2) produced by hematopoietic cells regulates intestinal inflammation. **(A)** Bone marrow (BM) recipient mice were irradiated as indicated in Section “Materials and Methods,” and chimeric mice were generated by caudal vein injection of WT or Fgl2^−/−^ mouse-derived BM cells. **(B–F)** After 8 weeks, mice were subjected to dextran sodium sulfate (DSS)-induced colitis and weight loss **(B)**, clinical score **(C)**, and colon length **(D)** were evaluated. Representative images and quantification **(E)** of histological analyses are shown (*n* = 10 for WT→WT; *n* = 9 for Fgl2^−/−^→WT). **(F)** On day 9 of DSS induction, colonic mucosal production of pro-inflammatory and regulatory cytokines was evaluated by enzyme-linked immunosorbent assay (*n* = 8 for WT→WT; *n* = 7 for Fgl2^−/−^→WT). Data are expressed as the mean ± SEM and were replicated in two independent experiments. The statistical significance of differences was determined by two-way analysis of variance with Bonferroni correction **(B,C)** and unpaired two-tailed Student’s *t*-test **(D–F)**. *P < 0.05, **P < 0.01, ***P < 0.001, n.s., not significant.

The above results indicated that the development of colitis is limited by Fgl2, which is expressed predominantly, if not exclusively, by colonic immune cell types from the hematopoietic compartment in colitis. Thus, we next aimed to ascertain whether hematopoietic cell-derived Fgl2 exerts protective effect against DSS-induced colitis. To this end, we generated two types of BM chimeric mice: one group consisted of lethally irradiated WT (CD45.1) mice engrafted with BM cells isolated from WT (CD45.2) littermates, yielding WT→WT BM chimeras; and the other group consisted of lethally irradiated WT (CD45.1) mice engrafted with BM cells isolated from their Fgl2^−/−^ littermates (CD45.2), yielding Fgl2^−/−^→WT BM chimeras, to knockout Fgl2 exclusively in all hematopoietic cells (Figure 3A; Figure S3A in Supplementary Material). As expected, 8 weeks after BM injection, mice harboring Fgl2^−/−^ BM cells expressed much lower levels of both mFgl2 and sFgl2 than those that received WT BM cells (Figures S3B,C in Supplementary Material). The mice were then subjected to DSS treatment. Significantly, Fgl2^−/−^→WT BM chimeric mice developed more severe colitis, with a phenotype similar to that of Fgl2^−/−^ mice (Figure 2), as evidenced by weight loss (Figure 3B), clinic scores (Figure 3C), colon shortening (Figure 3D), histopathological alterations (Figure 3E), and inflammatory cytokine levels in colonic tissues (Figure 3F). These results demonstrate that Fgl2 expression by hematopoietic cells is essential for controlling intestinal inflammation.

### Loss of Fgl2 Facilitates M1 Polarization, Whereas It Suppresses M2 Polarization

**Figure 4:**
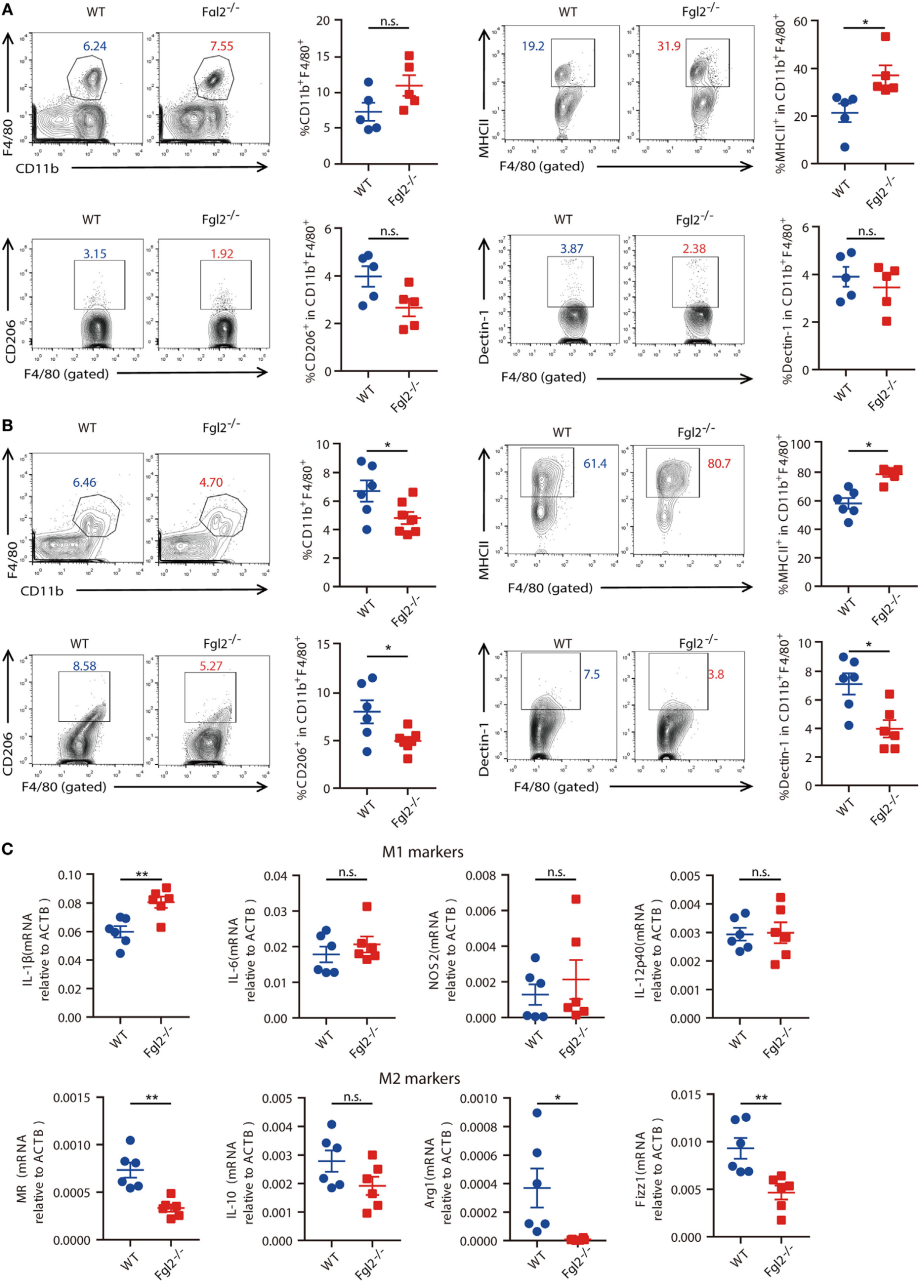
Fibrinogen-like protein 2 (Fgl2) is required for regulation of M1/M2 polarization in colitis. **(A,B)** On day 6 (*n* = 5 per group) **(A)** and day 9 (*n* = 6 for WT; *n* = 6–7 for Fgl2^−/−^) **(B)** of 2.5% dextran sodium sulfate (DSS) induction, the percentage of macrophages (CD11b^+^ F4/80^+^), M1 macrophages (MHCII^+^), and M2 macrophages (CD206^+^ or Dectin-1^+^) in colonic lamina propria (cLP) isolated from colitic WT and Fgl2^−/−^ mice was detected by flow cytometry. Numbers adjacent to the outlined areas indicate the percentages of the gated population in each group. **(C)** Macrophages in the cLP were isolated from WT and Fgl2^−/−^ mice on day 9 after DSS colitis induction. Expression of M1- and M2-associated genes was examined by real-time PCR (*n* = 6 per group). Data represent the mean ± SEM and are representative of three independent experiments. *P < 0.05, **P < 0.01, n.s., not significant (as determined by unpaired two-tailed Student’s *t*-test).

Next, we sought to further investigate the mechanism by which Fgl2 interferes with the progression of experimentally induced colitis. Given the crucial role of innate immune cells in both the onset and resolution of inflammation in DSS-induced colitis (10, 24, 25) and the regulatory functions of Fgl2 in various immune cells, we examined whether and how loss of Fgl2 impacts colonic innate immune cell infiltration. On day 6 of DSS induction, there were no differences in the percentages of colonic macrophages, neutrophils, and DCs between WT and Fgl2^−/−^ mice (Figure 4A; Figure S4A in Supplementary Material). Because activated macrophages can be polarized into M1 or M2 subtypes and modulating M1/M2 polarization can affect colitis severity (10), we wondered if macrophage polarization is perturbed in the absence of Fgl2. On day 6, the percentage of MHC-II-positive M1 macrophages in the colon was increased in Fgl2^−/−^ mice as compared with WT mice (Figure 4A), indicating that Fgl2 ablation leads to an accumulation of pro-inflammatory M1 macrophages in the colitic colon. This can explain the greater sensitivity of Fgl2^−/−^ mice to DSS-induced colitis. In contrast, Fgl2 deficiency only slightly decreased the percentage of colonic CD206 or Dectin-1-positive M2 macrophages, although the difference was not statistically significant (Figure 4A). Notably, at a later stage in colitis progression (day 9), the changes became more evident: loss of Fgl2 not only increased the proportion of M1 macrophages but also reduced the size of M2 populations (Figure 4B), suggesting that Fgl2 ablation promotes M1 polarization, whereas suppresses M2 polarization in DSS-induced colitis. On the other hand, the percentages of colonic neutrophils and DCs were unaffected by Fgl2 depletion until day 9 (Figure S4B in Supplementary Material).

M1 and M2 macrophages secrete distinct sets of cytokines (26). To clarify the role of Fgl2 in macrophage polarization, we sorted macrophages from the inflamed colon and analyzed cytokine production. As expected, Fgl2-deficient colonic macrophages isolated from DSS-treated mice produced higher levels of the M1 effector molecule IL-1β, but lower levels of the M2 polarization markers MR, Arg-1, and Fizz1 (Figure 4C) in DSS colitis than macrophages from DSS-treated WT mice.

These data indicate that in DSS-induced colitis, Fgl2 deficiency facilitates macrophage polarization towards pro-inflammatory M1 type, while it hinders beneficial M2 polarization, which likely, at least in part, amplifies the release of pro-inflammatory cytokines at mucosal sites and increases susceptibility to colitis.

It is currently unclear whether Fgl2 regulates macrophage polarization in a cell-intrinsic manner. To evaluate this, PEMs isolated from WT and Fgl2^−/−^ mice were treated with LPS and recombinant mouse IL-4 to induce M1 and M2 polarization, respectively. Upon LPS stimulation, PEMs from Fgl2^−/−^ mice secreted higher levels of IL-1β and -6 and nitric oxide synthase 2, indicating preferential differentiation into M1 macrophages (Figure S5A in Supplementary Material). In contrast, Fgl2-deficient PEMs showed reduced capacity for differentiation into M2 cells upon IL-4 stimulation, as determined by decreased expression of Arg1, MR, YM1, and IL-10 (Figure S5B in Supplementary Material). These results indicate that Fgl2 deficiency directly promotes the M1 phenotype, while suppressing the M2 phenotype, independent of intestinal inflammation.

### Fgl2 Deficiency Leads to the Development of CAC

**Figure 5:**
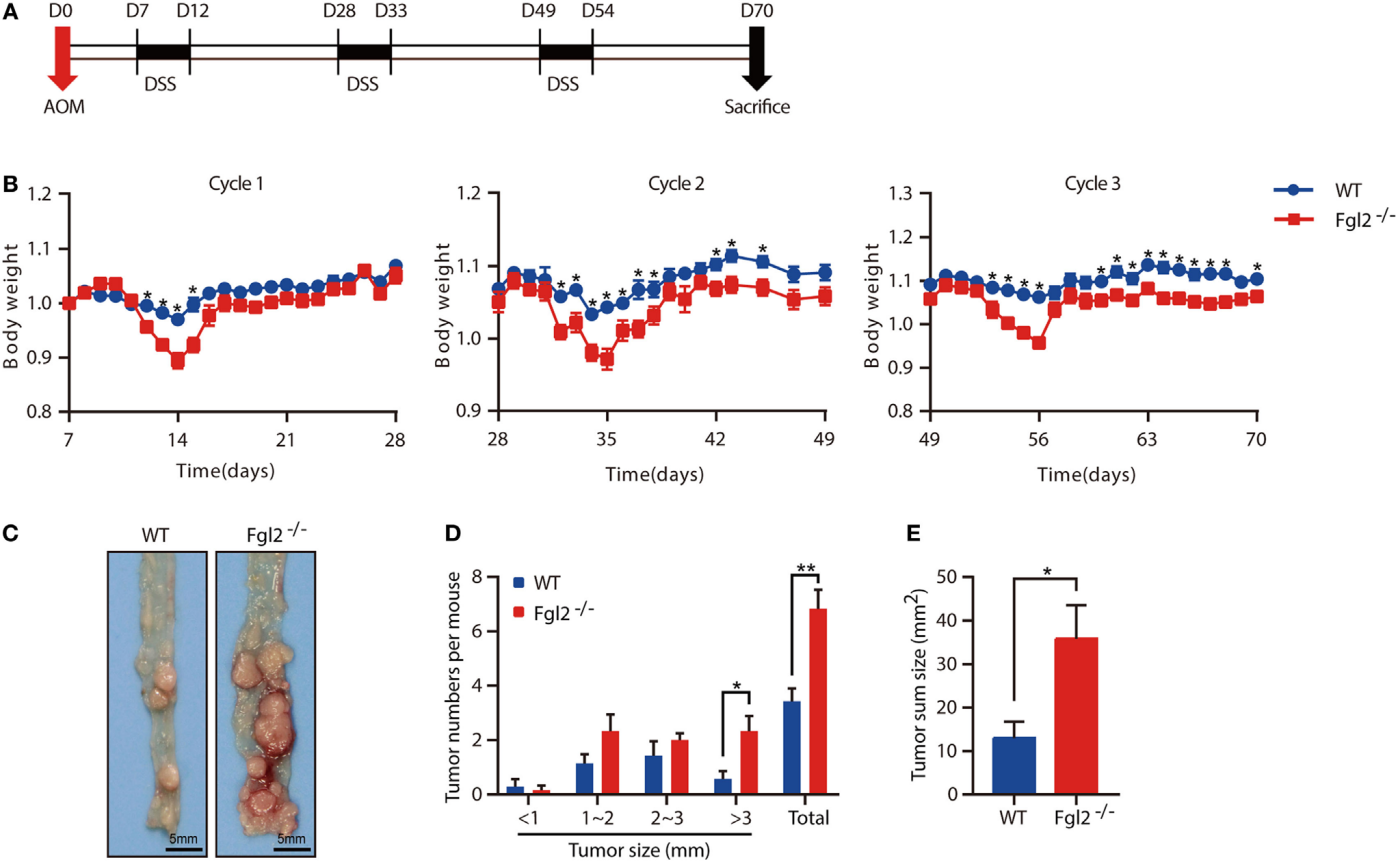
Fibrinogen-like protein 2 (Fgl2) deficiency promotes colitis-associated colorectal cancer (CAC). Fgl2^−/−^ mice (*n* = 7) and WT littermates (*n* = 8) were injected with azoxymethane (AOM) (10 mg/kg); 7 days later, mice were treated with three cycles of dextran sodium sulfate (DSS) (5 days of 1.8% DSS in water and 16 days of normal water). **(A)** Induction procedure for AOM/DSS-induced CAC in WT and Fgl2^−/−^ mice. **(B)** Body weight was monitored throughout the treatment regimen (*n* = 7–8 for WT, *n* = 6–7 for Fgl2^−/−^). **(C)** Representative images of colorectal tumors (*n* = 7 for WT, *n* = 6 for Fgl2^−/−^). **(D)** Number and size distributions of colorectal polyps (*n* = 7 for WT, *n* = 6 for Fgl2^−/−^). **(E)** Average tumor burden (*n* = 7 for WT, *n* = 6 for Fgl2^−/−^). Data are representative of three independent experiments. The statistical significance of differences was determined by two-way analysis of variance **(B)** with Bonferroni correction and unpaired two-tailed Student’s *t*-test **(D,E)**. *P < 0.05, **P < 0.01.

Colitis severity is known to influence the development of CAC (27). The observation that depletion of Fgl2 leads to exacerbated colitis prompted us to investigate whether and how Fgl2 deficiency affects the development of CAC. To this end, we induced CAC in WT and Fgl2^−/−^ mice with the AOM/DSS protocol (Figure 5A). In accordance with our observations in the colitis model, during each cycle of DSS challenge, Fgl2^−/−^ mice lost more body weight than their WT littermates (Figure 5B). Strikingly, we noticed that Fgl2^−/−^ mice had more tumors and a higher tumor load than WT mice (Figures 5C–E). Collectively, these results suggest that Fgl2 deficiency promotes CAC development.

## Discussion

Fibrinogen-like protein 2, a pivotal immune regulator, has been implicated in the pathogenesis of several inflammatory disorders (11, 14). However, the role of Fgl2 in IBD is not well understood but may provide valuable insight into IBD pathogenesis and clues to find novel therapeutic strategies. Here, we show that Fgl2 expression is significantly upregulated during DSS-induced mouse model of colitis, a widely used model that mimics the clinical features of human ulcerative colitis (22). This is consistent with recent reports in a 2,4,6-trinitrobenzene sulfonic acid-induced mouse model (28) (which resembles human CD) (22) and clinical evidence from patients with IBD (18). In addition, we demonstrate that Fgl2 exerts a protective effect by limiting intestinal inflammation and suppressing CAC. In this regard, Fgl2 overexpression in IBD may be the result of a feedback mechanism that is important but insufficient in and of itself to suppress inflammation. Thus, the use of recombinant Fgl2 may be an effective approach to inhibiting intestinal inflammation and tumorigenesis. To this end, the exact mechanisms that trigger and maintain Fgl2 expression in colitis should be clarified in future.

Fibrinogen-like protein 2 exists in a membrane-bound form with pro-coagulant activity and in a secreted form with immune regulatory functions (11). Notably, we observed a gradual increase in sFgl2 expression with the progression of colitis. Given that sFgl2 is readily detected, our results, together with previous findings (18), support the notion that Fgl2, especially sFgl2, may serve as a valuable and convenient biomarker for continuous monitoring of IBD progression and response to treatment in both animal research and clinical application. In addition to sFgl2, mFgl2 expression was upregulated in the inflamed colon, implying a role for this form in the modulation of intestinal inflammation. In terms of structure, mFgl2 and sFgl2 share a common fibrinogen-related domain that is responsible for the immune-regulatory effect of sFgl2. Currently, there is no evidence that mFgl2 acts to mount an immunologic response like sFgl2 does. It would be of interest to determine whether mFgl2 can limit immunopathological damage by fibrinogen-related domain-mediated immune regulatory activity and if so, its relative contribution in the control of intestinal inflammation.

Fibrinogen-like protein 2 has been found to be expressed in intestinal endothelial cells and infiltrating inflammatory cells in mucosal biopsy specimens of patients with IBD (18). Intriguingly, Fgl2 was mostly absent in colonic endothelial cells in our study. Possible reasons for these seemingly inconsistent results are that Fgl2 is expressed by endothelial cells during a chronic process that could not be detected in the DSS-induced acute colitis model, or that Fgl2 expression profiles differ between humans and rodents. We also demonstrated that Fgl2 is expressed by various inflammatory cell types. Furthermore, we proved that Fgl2 function in colitis is related to its expression in BM-derived cells. To date, experimental evidence is still lacking to address which cell type drives Fgl2 expression in intestinal inflammation, we found that colonic macrophages are the major source of both mFgl2 and sFgl2 in colitis. A recent report implied that Tregs and effector T cells control intestinal homeostasis through expression of Fgl2 in a T cell-induced colitis model (29). Similarly, we propose here that in the context of inflammation, colonic macrophages may through express Fgl2 partially restore intestinal homeostasis.

Mechanically, we paid special attention to macrophages, because they are among the most numerous leukocytes in the colon and are key mediators of the initiation and resolution of intestinal inflammation by being activated into either the M1 or the M2 phenotype (30, 31). In a mouse model of colitis and in patients with IBD, M1 macrophages were shown to infiltrate the cLP, shifting the balance in the macrophage pool toward a pro-inflammatory population and undermining epithelial integrity *via* the secretion of corresponding cytokines, ultimately leading to intestinal inflammation in IBD (32, 33). We also observed a constant pool of M1 macrophages within the lamina propria of inflamed gut tissue that was unexpectedly increased by loss of Fgl2, with concomitant aggravation of colitis. On the other hand, M2 macrophages are enriched in IBD animal models and clinical specimens, and this enrichment is inversely correlated with disease severity (10, 30, 32). It was previously shown that Fgl2 can increase the percentage of M2 macrophages in the tumor microenvironment (34). Interestingly, during the late stage of DSS-induced acute colitis, colonic M2 accumulation was decreased in the absence of Fgl2. Moreover, Fgl2-deficient macrophages exhibited an enhanced M1 response and impaired M2 phenotype. Thus, at the early stage of DSS-induced colitis, increased M1 polarization and activation by Fgl2 depletion may contribute to the exacerbated colitis, whereas during the late phase of DSS colitis, suppression of M2 polarization may be additionally responsible for colitis aggravation. However, we cannot rule out the possibility that Fgl2 deficiency also affects the number or activity of other leukocytes, such as Tregs and effector T cells, as reported by others (11).

We showed that Fgl2 directly regulates macrophage polarization and function in a cell-intrinsic manner, as loss of Fgl2 increased the expression of M1-associated genes and cytokines in LPS-stimulated PEMs, while reducing that of M2-associated genes and cytokines in PEMs activated by IL-4. Macrophage polarization is important for the initiation and progression of various pathological conditions, including infection, inflammation, atherosclerosis, obesity, asthma, sepsis, and tumors (35). Our findings therefore indicate that Fgl2 may act as a potential therapeutic target for these diseases owing to its role in regulating macrophage polarization. A network of signaling molecules, transcription factors, epigenetic mechanisms, and post-transcriptional regulators orchestrate macrophage polarization (36, 37); our results provide evidence that Fgl2 also performs this function, although additional studies are needed to clarify the precise molecular mechanisms.

In summary, our results presented herein point to Fgl2 as a novel protective molecule in the pathogenesis of IBD. We found that Fgl2 is critical for limiting DSS-induced colitis and subsequent CAC development through its effects on macrophage polarization and might serve as a biomarker and/or therapeutic target in the treatment of IBD and other inflammatory diseases.

## Ethics Statement

All animal experiments were approved by the Institutional Animal Care and Use Committee of Third Military Medical University, China.

## Author Contributions

BZ, BG, YZ, JZ, and YF designed the experiments; YZ, JZ, YF, LC, LZ, FY, HZ, XW, XH, and CS acquired the data; YZ, JZ, YF, and LC analyzed and interpreted the data; YZ and JZ wrote the manuscript; BZ, BG, YW, and QL revised the manuscript; and all authors read and approved the manuscript for publication.

## Conflict of Interest Statement

The authors declare that the research was conducted in the absence of any commercial or financial relationships that could be construed as a potential or reproduction is permitted which does not comply with these terms.
