# Supplementary material for: Control of Intestinal Inflammation, Colitis-Associated Tumorigenesis, and Macrophage Polarization by Fibrinogen-Like Protein 2
Source: Front Immunol. 2018 Jan 30;9:87. doi: 10.3389/fimmu.2018.00087 (PMC5797584; doi:10.3389/fimmu.2018.00087)
Supplement: Supplementary file 1 [file Table_1.DOCX]

Supplementary Material

**Control of intestinal inflammation, colitis-associated tumorigenesis, and macrophage polarization by Fgl2**

**Ying Zhu, Jie Zhou, Yi Feng, Liying Chen, Longhui Zhang, Fei Yang, Haoran Zha, Xinxin Wang, Xiao Han, Chi Shu, Yisong Y. Wan, Qi-Jing Li, Bo Guo, Bo Zhu***

*** Correspondence:** Bo Zhu; E-mail: b.davis.zhu@gmail.com

# Supplementary Table

**Supplementary Table 1**. **The primer pairs used in the study**

| Gene | Primer forward | Primer reverse |
| --- | --- | --- |
| IL-1β | 5’-gcaactgttcctgaactcaact-3’ | 5’-atcttttggggtccgtcaact -3’ |
| IL-6 | 5’-tagtccttcctaccccaatttc-3’ | 5’-ttggtccttagccactccttc-3’ |
| NOS2 | 5’-gccaccaacaatggcaaca-3’ | 5’-cgtaccggatgagctgtgaatt-3’ |
| IL-12p40 | 5’-agacatggagtcataggctctg-3’ | 5’-ccattttccttcttgtggagca-3’ |
| MR | 5’-ctcgtggatctccgtgacac-3’ | 5’-gcaaatggagccgtctgtgc-3’ |
| IL-10 | 5’-gctcttactgactggcatgag-3’ | 5’-cgcagctctaggagcatgtg-3’ |
| Fizz1 | 5’- tcccagtgaatactgatgaga-3’ | 5’- ccactctggatctcccaaga-3’ |
| Ym1 | 5’-gggcatacctttatcctgag -3’ | 5’-ccactgaagtcatccatgtc -3’ |
| Arg1 | 5’-ctccaagccaaagtcttagag-3’ | 5’-aggagctgtcattagggacatc-3’ |
| ACTB | 5’-ggctgtattcccctccatcg -3’ | 5’-ccagttggtaacaatgccatgt -3’ |
