# Supplementary material for: Control of Intestinal Inflammation, Colitis-Associated Tumorigenesis, and Macrophage Polarization by Fibrinogen-Like Protein 2
Source: Front Immunol. 2018 Jan 30;9:87. doi: 10.3389/fimmu.2018.00087 (PMC5797584; doi:10.3389/fimmu.2018.00087)
Supplement: Supplementary file 2 [file Image_1.PDF]

Supplementary Material

Control of intestinal inflammation, colitis-associated tumorigenesis, and macrophage polarization by Fgl2

Ying Zhu, Jie Zhou, Yi Feng, Liying Chen, Longhui Zhang, Fei Yang, Haoran Zha, Xinxin Wang, Xiao Han, Chi Shu, Yisong Y. Wan, Qi-Jing Li, Bo Guo, Bo Zhu*

*** Correspondence:** Bo Zhu; E-mail: b.davis.zhu@gmail.com

Supplementary Figures

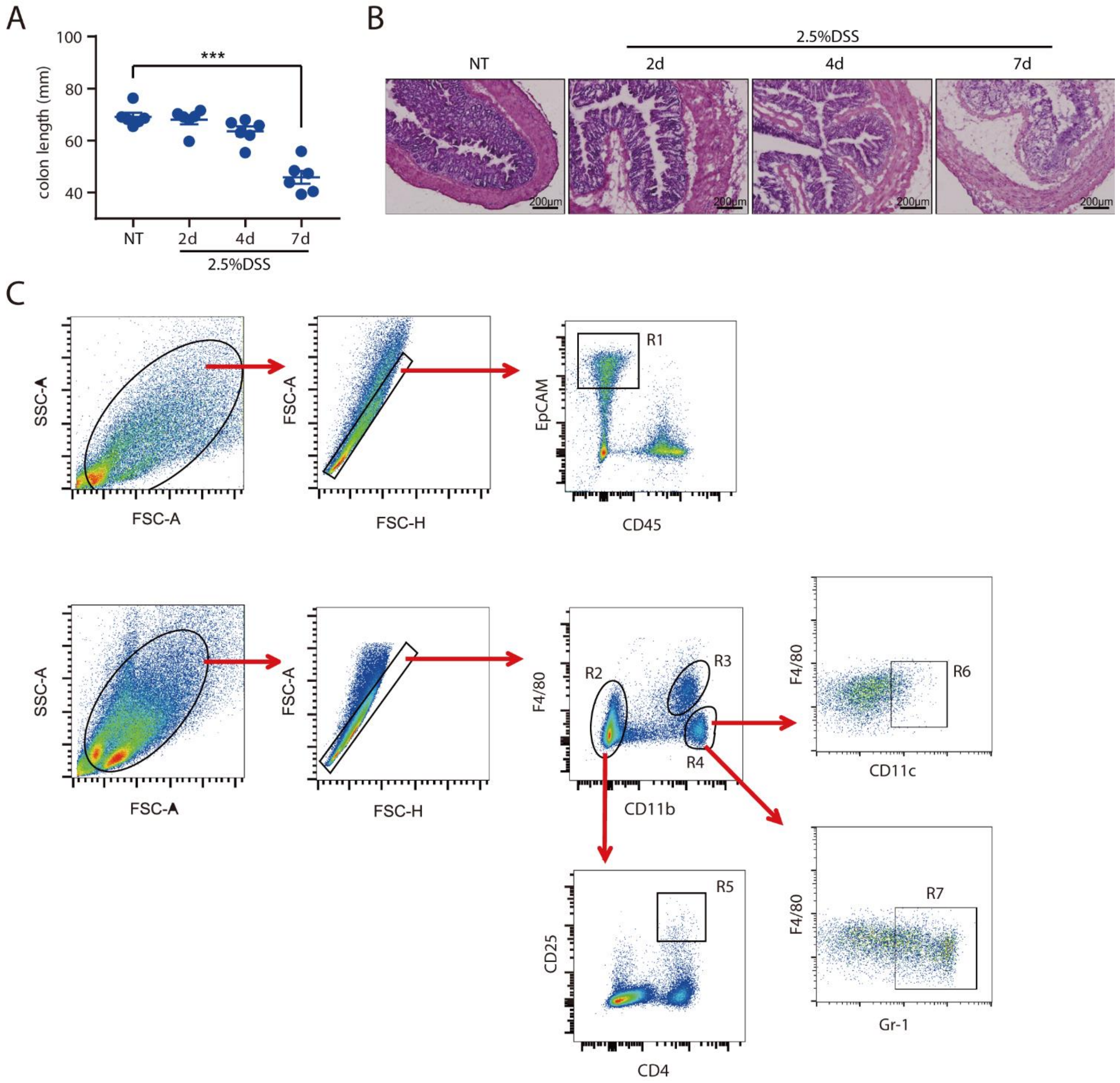

Supplementary Figure 1. Intestinal inflammation drives changes in Fgl2 expression in the colon. (A, B) Colitis was induced in C57BL/6 mice by 2.5% DSS treatment (n = 6). At indicated time points, mice were sacrificed, colon length was measured (A) and histological analysis was performed (B). (C) CD45^{-} EpCAM^{+} (R1), CD4^{+} CD25^{high} (R5), CD11b^{+} F4/80^{+} (R3), CD11b^{+} CD11C^{+} (R6), and CD11b^{+} Gr-1^{+} (R7) cells the colon of colitic C57BL/6 were sorted by flow cytometry on day 7. Quantitative data are expressed as the mean ± SEM and are representative of three independent experiments. *** $P < 0.001$ (as determined by one-way ANOVA with Bonferroni correction).

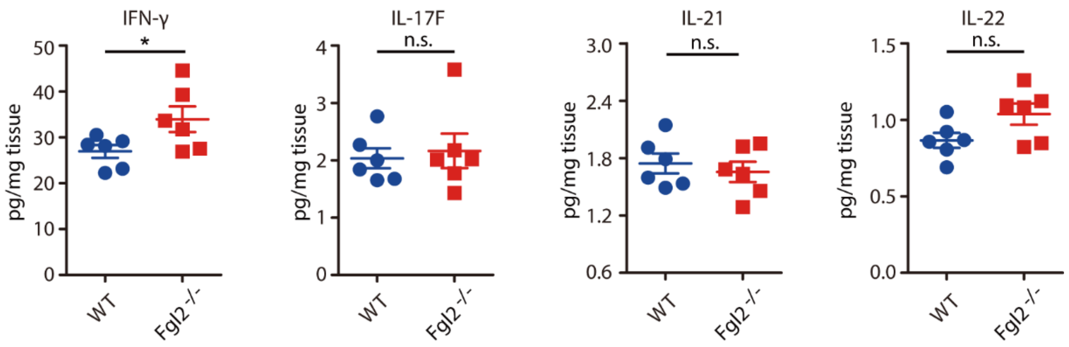

Supplementary Figure 2. Levels of colonic cytokine production after DSS treatment. Wild-type (WT) and Fgl2$^{-/-}$ littermates were treated as described in *Materials and Methods*. On day 9, the production of the pro-inflammatory cytokines IFN-γ, IL-17F, IL-21 and IL-22 in the colonic mucosa was evaluated by ELISA (n = 6 per group). Data are expressed as the mean ± SEM and were replicated in two independent experiments. * $P < 0.05$, n.s. = not significant (as determined by unpaired two-tailed Student's *t*-test).

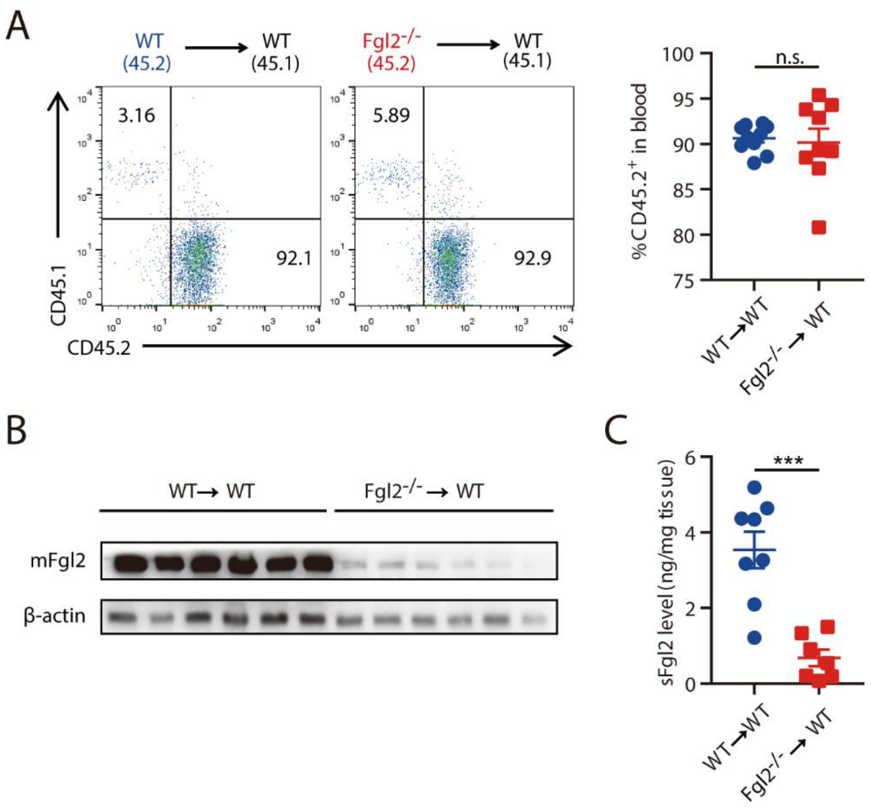

Supplementary Figure 3. Generation of WT and Fgl2$^{-/-}$ BM chimeric mice. (A) WT (CD45.1) mice were injected with BM cells from WT (CD45.2) or Fgl2$^{-/-}$ (CD45.2) mice after lethal irradiation; 8 weeks later, CD45^{+} allelic variants of peripheral blood leukocytes were detected by flow cytometry (n = 9 per group). (B, C) Evaluation of colonic mFgl2 and sFgl2 expression by western blotting (B) and ELISA (C) (n = 8 for WT→WT; n = 7 for Fgl2$^{-/-}$→WT). Data represent mean ± SEM and are representative of three independent experiments. *** $P < 0.001$, n.s. = not significant (as determined by unpaired two-tailed Student's *t*-test).

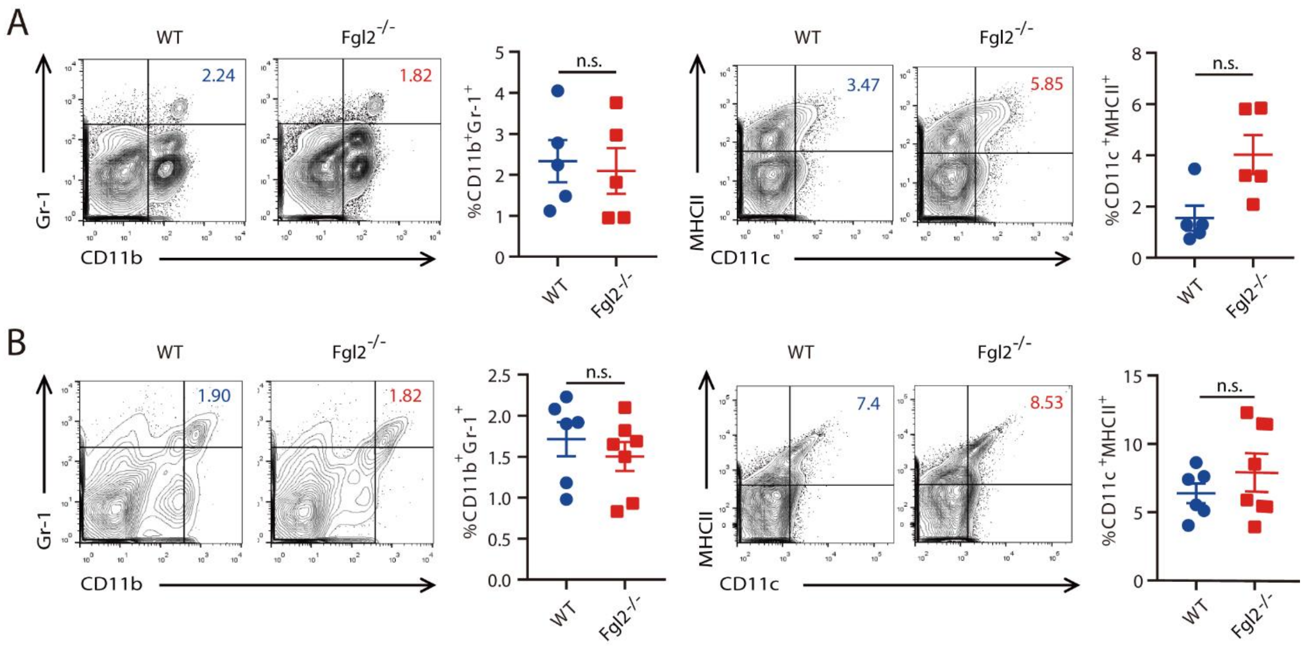

Supplementary Figure 4. Percentages of neutrophils and mature DCs among cLP from WT and Fgl2$^{-/-}$ mice. (A, B) On day 6 (A) and day 9 (B) after 2.5% DSS treatment, the neutrophil (CD11b^{high} Gr-1^{high}) and mature DC (CD11c^{+} MHC-II^{+}) fractions were detected by flow cytometry (day6: n = 5 per group; day9: n = 6 for WT; n = 7 for Fgl2$^{-/-}$). Numbers adjacent to outlined areas indicate percentages of the gated population in each group. Data are representative of three independent experiments. Error bars indicate SEM. n.s. = not significant (as determined by unpaired two-tailed Student's t-test).

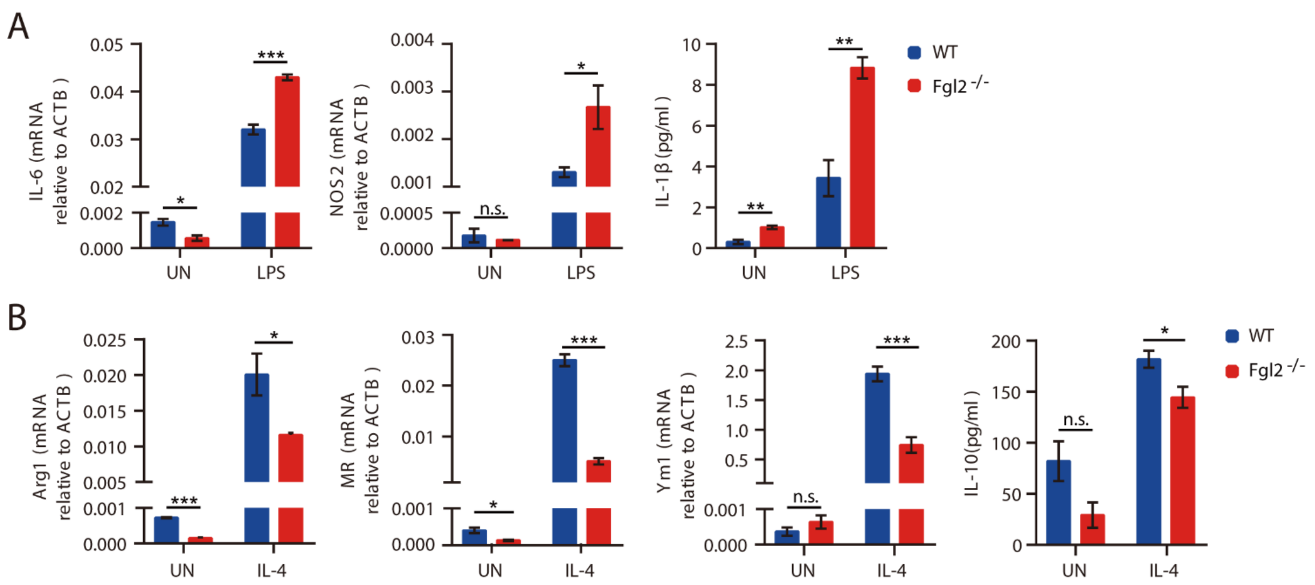

Supplementary Figure 5. Fgl2 regulates macrophage polarization and function *in vitro*. (A, B) PEMs isolated from WT and Fgl2$^{-/-}$ mice were cultured and stimulated *in vitro* with 100 ng/ml LPS (A) or 20 ng/ml recombinant mouse IL-4 (B) for 12 or 48 h. Expression of indicated genes and cytokines was examined by qRT-PCR or ELISA (n = 3 per group). Data represent the mean ± SEM of three independent experiments. * $P < 0.05$, ** $P < 0.01$, *** $P < 0.001$, n.s. = not significant (as determined by unpaired two-tailed Student's *t*-test).
